# Correction: Pest susceptibility, yield and fiber traits of transgenic cotton cultivars in Multan, Pakistan

**DOI:** 10.1371/journal.pone.0240391

**Published:** 2020-10-05

**Authors:** Haider Karar, Muhammad Amjad Bashir, Muneeba Haider, Najeeba Haider, Khalid Ali Khan, Hamed A. Ghramh, Mohammad Javed Ansari, Çetin Mutlu, Suilman Mohammad Alghanem

The affiliation for the ninth author is incorrect. The correct affiliation is not indicated. Suilman Mohammad Alghanem is not affiliated with #6 but with: Biology Department, Faculty of Sciences, University of Tabuk, Tabuk, KSA.
