# Correction: Correction: Pest susceptibility, yield and fiber traits of transgenic cotton cultivars in Multan, Pakistan

**DOI:** 10.1371/journal.pone.0242180

**Published:** 2020-11-05

**Authors:** The PLOS ONE Staff

There are errors in the correction published on October 5, 2020. The publisher apologizes for the error. The correct text is:

The ninth author's name is spelled incorrectly. The correct name is: Suliman Mohammad Alghanem. Additionally, the affiliation for the ninth author is incorrect. The correct affiliations are as follows:

Haider Karar^1^, Muhammad Amjad Bashir^2^, Muneeba Haider^3^, Najeeba Haider^3^, Khalid Ali Khan^4,5,6^, Hamed A. Ghramh^4,5,6^, Mohammad Javed Ansari^7^, Çetin Mutlu^8^, Suliman Mohammad Alghanem^9^

**1** Mango Research Institute, Multan, Pakistan, **2** Department of Plant Protection faculty of Agricultural Sciences, Ghazi University Dera Ghazi Khan Punjab, Dera Ghazi Khan, Pakistan, **3** Muhammad Nawaz Shareef University of Agriculture, Multan, Pakistan, **4** Research Center for Advanced Materials Science (RCAMS), King Khalid University, Abha, Saudi Arabia, **5** Unit of Bee Research and Honey Production, Faculty of Science, King Khalid University, Abha, Saudi Arabia, **6** Biology Department, Faculty of Science, King Khalid University, Abha, Saudi Arabia, **7** Department of Botany, Hindu College Moradabad, Moradabad, Uttar Pradesh, India, **8** Department of Plant Protection, Harran University, Şanlıurfa, Turkey, **9** Biology Department, Faculty of Science, Tabuk University, Tabuk, Saudi Arabia
